# Seed biology – from lab to field

**DOI:** 10.1093/jxb/erx021

**Published:** 2017-02-24

**Authors:** Steven Penfield

**Affiliations:** 1 Department of Crop Genetics, John Innes Centre, Norwich Research Park, Norwick, NR4 7UH, UK

**Keywords:** Fertilization, gamete production, germination, seed dormancy, seed longevity, seed maturation, seed variation.

**Seed biology is a highly dynamic field of plant science with several areas advancing at pace over the past two years. The reviews in this special issue of *Journal of Experimental Botany* provide an incisive commentary on these advances, which are significant not just for our understanding of basic seed development and behaviour, but also industrial application.**

Recent progress in research on seed biology can be divided into two major domains. The first of these covers fertilization biology, gamete production, fertilization and the initiation of development of the major seed tissues. Significant steps forward are being made in our understanding of the genetic, epigenetic and morpho-mechanical control of these areas. It is now becoming clearer that seed developmental processes are also finely intertwined with other mechanisms that govern seed behaviour, such as seed dormancy, germination and longevity.

A second major theme is a move away from a deterministic view of seed science where all seeds are considered equivalent to understanding the importance of variation in seeds and its role in selective processes. This means combining fields of ecology with molecular genetics and modelling to understand the role of environmental signalling and developmental processes in generating behavioural variation among and within populations of seeds. These rapidly evolving developments in seed biology are highlighted throughout the reviews in this issue.

## Tissue to tissue communication

Communication between tissues is a newly developing topic in seed research, and it is now clear that sharing signals between tissues is a central part of reproductive biology, starting during germline development and gametogenesis through seed development, dormancy and germination.

Wang and Köhler (2017) describe the dramatic whole-genome changes in DNA methylation that take place during the production of microspores and megaspores, and the key role that transposable elements play in directing changes in DNA methylation to different loci during reproductive development, especially in gene imprinting in the endosperm (Gehring *et al.*, 2009). During gametogenesis an emerging theme is the role of the vegetative cell in pollen development and the central cell in megaspore development in providing small RNAs to the male and female gametes that play a key role in regulating gene expression by limiting the expression of transposable elements (Ibarra *et al.*, 2012; Martinez *et al.*, 2016). This mechanism is also central to imprinted gene expression in the endosperm post-fertilization, where differentially methylated regions associated with transposable elements give rise to loci where either the maternal or paternal allele is preferentially expressed (Rodrigues and Zilberman, 2015). A key role of paternally expressed genes is the regulation of auxin levels in the gamete and this is important for the normal control of endosperm proliferation by fertilization (Figueiredo *et al.*, 2015). In another recent contribution from the Kohler lab, Figueiredo *et al.* (2016) reveal a further role for this endospermic auxin pulse in the initiation of seed coat development. Together, this establishes the central role of endospermic auxin in the control of seed development.

Elsewhere in this issue, Ingram (2017) describes the production of a mobile signal by endospermic *AGAMOUS LIKE 62* *(AGL62*) expression in Arabidopsis, which turns out to be the export of auxin referred to earlier. Auxin also plays a key role in the initiation of fruit development, and in many species application of exogenous auxin leads to parthenocarpic fruit development, underlying the important role of auxin in coordinating fruit and seed development.

During the development of the seed, Ingram (2017) describes how the embryo and endosperm compete for space within the developing integuments. This is likely to be important in dormancy regulation because the biomechanical constraints described during germination (Steinbrecher and Leubner-Metzger, 2017) are probably established by this competitive process during seed maturation. Much variation in seed morphology in angiosperms is determined by the ratio of embryo and endosperm in the mature seed. Ingram describes how embryo-dominated seeds rely on a complex of two bHLH transcription factors, INDUCER OF CBF EXPRESSION1 (ICE1) and ZHOUPI (ZOU), to promote endospermic cell death, thereby making way for embryo growth within the integuments (Fourquin *et al.*, 2016). The seed environment is interesting because it permits a kind of mechanical communication between embryo and endosperm that controls the final balance of the two tissues in the mature seed. ZOU has an additional role in providing an as yet unknown signal to the embryo which is necessary for cuticle formation (Xing *et al.*, 2013). This cuticle may also be important after maturity as the embryo and endosperm swap hormonal signals that control seed dormancy and germination.

This latter aspect is discussed in detail by Chahtane *et al.* (2017) who focus on mechanisms of dormancy imposition and loss. Of central importance in dormancy imposition is the endosperm. This synthesizes and transports abscisic acid (ABA) to the embryo to promote quiescence. The authors then continue to discuss more general aspects of the mechanism behind dry afteripening in seeds and the role of reactive oxygen species. One producer of reactive oxygen species in seeds is the respiratory burst oxidase RBOHD, which is also expressed in the endosperm where it plays a role in the ABA response (Penfield *et al.*, 2006).

## Environment, maturation and control of processes post-shedding

Tissue to tissue communication in the control of behavioural plasticity is also a theme, with Penfield and MacGregor (2017) focusing on mechanisms by which environmental signals control seed dormancy. While information on fertilization status is communicated from the endosperm to other parts of the ovule and fruit, environmental information is communicated from fruit to seed by means of regulation of *FLOWERING LOCUS T* expression (Chen *et al.*, 2014). These studies shed light as to the impact of extrinsic variability from the environment’s role in impacting the behaviour of seed populations, and the key role of the mother plant in shaping progeny seed dormancy characteristics at the population level. Added to this Mitchell *et al.* (2017) also point to intrinsic cellular and tissue-based processes for generating variation in seed behaviour both within and across populations.

The control of seed maturation is covered by both Leprince *et al.* (2017) and Carbonero *et al.* (2017), who both describe the role of ABA signalling in the control of seed metabolism, reserve accumulation and desiccation tolerance. After dealing with seed development and dormancy induction, four contributions focus on the control of dormancy and/or germination post-shedding.

Finch-Savage and Footitt (2017) provide an excellent overview of their innovative molecular ecology approach to seed dormancy cycling. They show how gene expression and mutant studies together point to MOTHER OF FT AND TFL1 (MFT) as a likely key regulator of life history variation in seeds (Vaistij *et al.*, 2012; Footitt *et al.*, 2013). Systems-based approaches are increasingly being employed to untangle complex traits in seeds. Gene co-expression networks have been used to identify genetic regulators of each seed dormancy (Bassel *et al.*, 2011) and seed maturation (see Righetti *et al.*, 2015; Leprince *et al.*, 2017). The cellular basis of dormancy and germination has also begun to be uncovered. Whole-embryo cellular resolution imaging and localization has identified the cells of the radicle to be the site where germination is initiated in Arabidopsis (Bassel *et al.*, 2014; Mitchell *et al.*, 2017). Disparity between non-intuitive radicle-derived gene expression and observed growth (Sliwinska *et al.*, 2009) is explained by cellular resolution mechanical models of embryo growth. The future will undoubtedly see integration of embryo-based models for germination control and endosperm-based models for dormancy alleviation which probably explain much post-imbibition activity in seeds with coat-imposed physiological dormancy.
